# Imidazo[1,2‐b]Pyrazole‐1,2,4‐oxadiazole Linked 1,2,3‐Triazoles: Synthesis, Antibacterial, Biofilm, and In Silico Studies

**DOI:** 10.1002/cbdv.71816

**Published:** 2026-10-12

**Authors:** Suresh Kumar Murahari, Kavitha Siddoju

**Affiliations:** ^1^ Department of Chemistry Chaitanya (Deemed to Be University) Hyderabad India

**Keywords:** antibacterial, antibiofilm, molecular docking, 1,2,3‐triazole, 1,2,4‐oxadiazole, Imidazo[1,2‐b]pyrazole

## Abstract

The growing incidence of bacterial resistance, especially in gram‐positive infections, emphasizes how urgently new antibacterial medicines must be developed. This study presents the rational design and synthesis of a series of fused imidazo[1,2‐b]pyrazole–1,2,4‐oxadiazole–1,2,3‐triazole hybrids (**8a–8n**) employing a multi‐step synthetic approach that includes oxidative cyclisation, amidoxime formation, Vilsmeier‐mediated cyclodehydration, and Cu(I)‐catalyzed azide–alkyne cycloaddition (CuAAC). The synthesized compounds were assessed for their in vitro antibacterial efficacy against *Bacillus subtilis*, *Staphylococcus aureus*, and *Staphylococcus epidermidis*. Multiple derivatives displayed notable antibacterial activity, with compounds **8j** and **8l** identified as the most effective (MIC = 1.56 µg/mL), exhibiting antibacterial efficacy comparable to or surpassing that of the reference medication dicloxacillin. Biofilm inhibition tests further indicated that compounds **8j** and **8l** exhibit substantial antibiofilm action. Molecular docking analyses targeting penicillin‐binding protein (PDB ID: **1MWT**) demonstrated advantageous binding affinities, with compound 8k displaying the most favorable binding score (−9.10 kcal/mol), corroborating the experimental results. Structure–activity relationship (SAR) research revealed that the inclusion of electron‐withdrawing substituents, especially dihalogen groups, markedly increases antibacterial efficacy. These results underscore the therapeutic potential of fused imidazo[1,2‐b]pyrazole–1,2,4‐oxadiazole–1,2,3‐triazole hybrids as effective antibacterial and antibiofilm agents against gram‐positive bacterial infections.

## Graphical abstract

A collection of innovative imidazo[1,2‐b]pyrazole–1,2,4‐oxadiazole–1,2,3‐triazole hybrids was conceived via the molecular hybridization of bioactive pharmacophores and produced via a CuAAC methodology. The synthesized compounds exhibited strong antibacterial and antibiofilm properties against gram‐positive bacteria. Compounds **8j** and **8l** had the most potent antibacterial efficacy (MIC = 1.56 µg/mL), surpassing dicloxacillin. Investigations into structure–activity relationships demonstrated that 3,5‐dihalogen substitution markedly improved biological efficacy, designating these hybrids as potential antibacterial lead compounds.

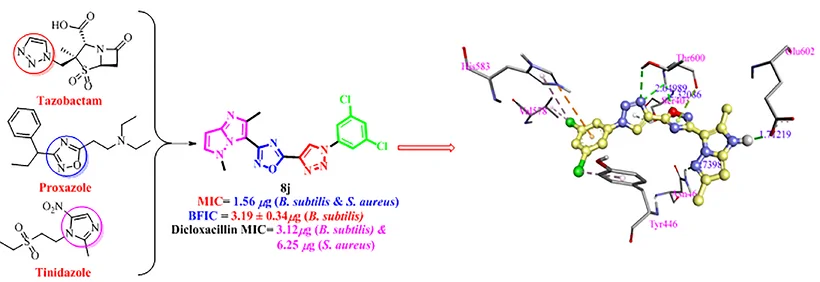

## 1 Introduction

Bacterial infections remain a significant hazard to worldwide public health, resulting in ailments that vary from minor symptoms like pharyngitis and urinary tract infections (UTIs) to severe diseases such as meningitis and pneumonia [1, 2]. In addition to acute infections, pathogenic bacteria induce chronic health difficulties by producing strong toxins that harm host tissues and organs [3]. Furthermore, mounting data indicates that the alteration of gut microbiota by pathogenic or opportunistic microorganisms correlates with the onset of digestive, metabolic, and neuropsychiatric diseases [4]. Gram‐positive bacteria are prevalent colonizers of the skin and mucosal surfaces in humans and animals among bacterial diseases [5]. Their pathogenicity is facilitated by a varied array of virulence factors that enhance adhesion, tissue invasion, immune evasion, and biofilm formation, allowing for infections that range from superficial skin conditions to severe systemic illnesses, including endocarditis, osteomyelitis, and sepsis [6].

In recent years, 1,2,3‐triazoles and their hybrid derivatives have become significant pharmacophores in medicinal chemistry due to their extensive range of biological activities and advantageous pharmacokinetic properties. The 1,2,3‐triazole ring functions as a bioisostere of the amide bond, demonstrating similar spatial orientation and electrical properties, therefore improving molecule stability and target binding affinity [7, 8]. As a result, many 1,2,3‐triazole compounds have been synthesized and documented to exhibit a range of pharmacological actions, including antitubercular [9, 10], antifungal [11, 12], anticancer [13, 14], and antibacterial characteristics [15, 16]. Several triazole‐containing compounds have exhibited significant action against gram‐positive bacterial pathogens, underscoring their potential as frameworks for the creation of new antibacterial medicines [17]. Alongside their advantageous bioisosteric characteristics, 1,2,3‐triazole derivatives can establish various noncovalent interactions, such as hydrogen bonding, π–π stacking, and dipole interactions, thereby improving aqueous solubility, molecular recognition, and binding affinity to biological targets [18]. Moreover, some clinically licensed antibacterial medicines, like Tazobactam and Cefatrizine, feature the 1,2,3‐triazole scaffold, highlighting its therapeutic significance (Figure 1) [19, 20]. Similarly, imidazole and fused imidazole ring complexes are esteemed structural motifs in medicinal chemistry due to their significant polarity, advantageous hydrogen‐bonding potential, and diverse pharmacological properties [21, 22, 23]. Heterocyclic frameworks are extensively utilized in the development of antimicrobial agents, with clinically significant imidazole‐containing drugs such as metronidazole, tinidazole, ornidazole, and nitrosoimidazole derivatives exhibiting broad‐spectrum antibacterial efficacy, especially against anaerobic bacterial infections [24].

**FIGURE 1:**
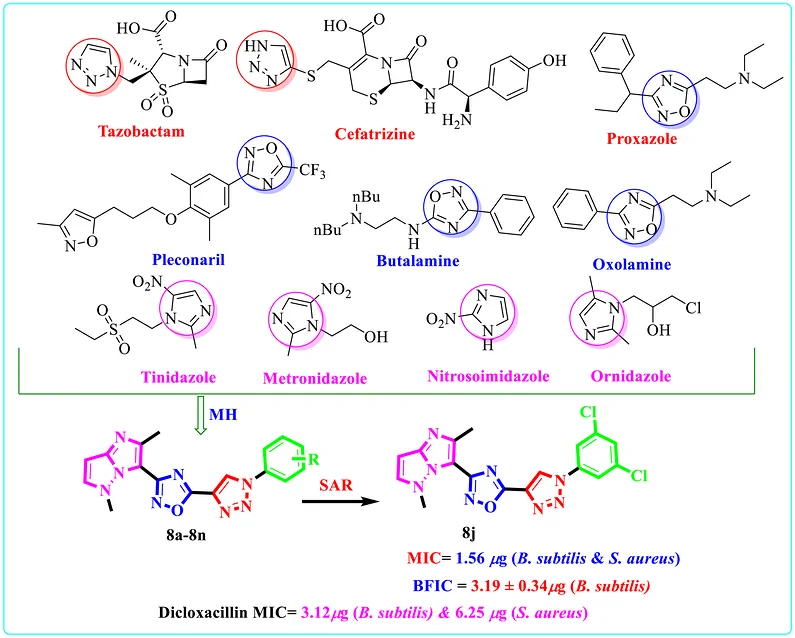
Examples pyrazole–oxadiazole–triazole containing drugs in market.

1,2,4‐Oxadiazoles represent a significant category of heterocyclic frameworks in medicinal chemistry due to their structural adaptability, advantageous physicochemical characteristics, and varied biological activities, rendering them essential components in contemporary drug discovery [25]. Numerous clinically significant medicines include the 1,2,4‐oxadiazole moiety, underscoring their pharmacological importance. Pleconaril functions as an inhibitor of picornavirus replication and has been studied for the treatment of respiratory viral infections [26, 27]. Oxolamine is commonly utilized as an antitussive medication that mitigates irritation of sensory nerve receptors in the respiratory system. Proxazole demonstrates analgesic and anti‐inflammatory effects and is utilized in treating functional gastrointestinal disorders [28], whereas Butalamine acts as a vasodilator and smooth muscle relaxant [29]. These examples illustrate the extensive therapeutic potential of the 1,2,4‐oxadiazole scaffold and endorse its ongoing investigation in the creation of innovative bioactive compounds.

The well‐established antibacterial potential of 1,2,3‐triazoles, 1,2,4‐oxadiazoles, and fused imidazole scaffolds has inspired the development of hybrid molecules that integrate these pharmacophores into a single molecular framework (Figure 1) [30, 31, 32]. Utilizing the molecular hybridization approach, we devised and synthesized an innovative series of fused imidazo[1,2‐b]pyrazole–1,2,4‐oxadiazole–1,2,3‐triazole hybrids through a Cu(I)‐catalyzed azide–alkyne 1,3‐dipolar cycloaddition (CuAAC) involving an alkyne intermediate and diverse aryl azides (Scheme 1). Recent studies have further demonstrated that strategic halogen substitution and heterocyclic functionalization can modulate molecular electronic properties, structural stability, target‐binding interactions, and biological activity, supporting the importance of substituent‐driven optimization in the design of biologically active heterocyclic scaffolds [33, 34, 35].

**SCHEME 1:**
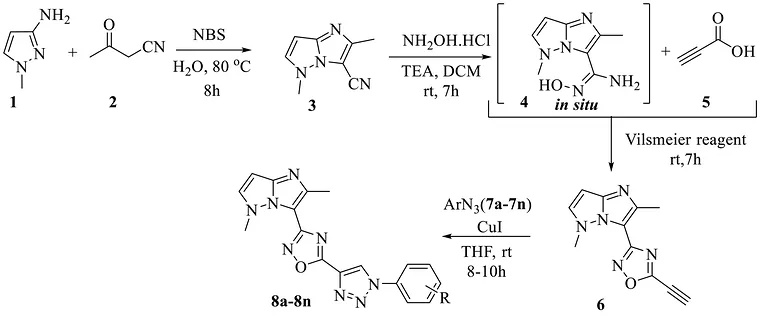
Synthesis of imidazo[1,2‐b]pyrazole‐1H‐1,2,3‐triazole‐1,2,4‐oxadiazoles (**8a‐8n**).

The novelty of the present study lies in integrating imidazole, 1,2,4‐oxadiazole, and 1,2,3‐triazole pharmacophores within a single hybrid molecular framework, thereby providing a structurally distinct platform for antibacterial lead development. Compared with compounds containing individual imidazole‐, oxadiazole‐, or triazole‐based pharmacophores, this hybrid architecture incorporates multiple heteroatom‐rich functionalities that may provide complementary noncovalent interactions and modulate molecular polarity, geometry, and target recognition. The observed activity of derivatives **8j** and **8l** supports the potential of this integrated scaffold; however, the individual contribution or synergistic effect of each heterocycle requires further comparative studies.

The synthesized compounds were subsequently assessed for their in vitro antibacterial activity against Gram‐positive bacteria and tested for in vitro antibiofilm efficacy. Furthermore, in silico molecular docking analyses were conducted to examine their binding interactions with the target protein and to corroborate the reported antibacterial efficacy.

## 2 Results and Discussion

Scheme 1 illustrates an effective synthesis technique for the preparation of fused imidazo[1,2‐b]pyrazole–1,2,4‐oxadiazole–1,2,3‐triazole hybrids via a series of meticulously planned transformations. Initially, 3‐aminopyrazole (**1**) was reacted with the activated nitrile‐containing ketone (**2**) in the presence of N‐bromosuccinimide (NBS) in an aqueous medium at 80°C, yielding intermediate **3** through an oxidative cyclisation process. NBS facilitates intramolecular ring closure, resulting in the synthesis of a fused imidazo[1,2‐b]pyrazole scaffold with a nitrile group. The reaction progressed seamlessly and concluded within 8 h, indicating excellent substrate compatibility and synthetic efficacy. Intermediate **3** was subsequently transformed into the equivalent amidoxime (**4**) through treatment with hydroxylamine hydrochloride (NH_2_OH·HCl) in the presence of triethylamine (TEA) in dichloromethane. This reaction entails the nucleophilic addition of hydroxylamine to the nitrile group, thus producing the amidoxime intermediate. The amidoxime was generated in situ, negating the necessity for isolation and purification, therefore streamlining the synthetic process and enhancing the overall efficiency of the reaction sequence.

The in situ produced amidoxime intermediate 4 was then reacted with the suitable carboxylic acid derivative 5 under Vilsmeier reagent conditions to yield the equivalent 1,2,4‐oxadiazole‐linked intermediate (6) via an effective cyclodehydration reaction. The Vilsmeier reagent activates the carboxylic acid group, boosting intermolecular cyclisation and enabling the synthesis of the 1,2,4‐oxadiazole ring. This step is crucial to the synthetic process, since the insertion of the oxadiazole moiety increases molecular stiffness, extends the conjugated heterocyclic framework, and offers an appropriate platform for further functionalization.

Intermediate 6, featuring a terminal alkyne group, was then exposed to Cu(I)‐catalyzed azide–alkyne cycloaddition (CuAAC) with several substituted aryl azides (ArN_3_) in THF at room temperature. This rapid “click chemistry” reaction produced the required fused imidazo[1,2‐b]pyrazole–1,2,4‐oxadiazole–1,2,3‐triazole hybrids (**8a–8n**) in good to outstanding yields within 8‐−10 h. The reaction demonstrated remarkable regioselectivity and extensive functional group tolerance, facilitating the integration of various aryl substituents without diminishing reaction efficiency. All the newly synthesized compounds were confirmed by ^1^H‐NMR, ^13^C‐NMR, and mass spectrometry (see Supporting Information). The synthetic strategy offers a direct and adaptable method for structural diversification, enabling the swift creation of a targeted library of hybrid molecules. The effective incorporation of three pharmacologically significant heterocyclic pharmacophores—imidazo[1,2‐b]pyrazole, 1,2,4‐oxadiazole, and 1,2,3‐triazole—into a unified molecular structure underscores the efficacy of the molecular hybridization strategy in creating biologically active antibacterial agents.

### 2.1 Antibacterial Activity

The antibacterial efficacy of compounds **8a–8n** was assessed against the gram‐positive bacterial strains *Bacillus subtilis*, *Staphylococcus aureus*, and *Staphylococcus epidermidis*, with the findings summarized in Table 1 [36]. Gram‐positive bacteria were chosen for the initial biological assessment due to the clinical significance of species like *S. aureus*, *S. epidermidis*, and *B. subtilis*, which are notable gram‐positive pathogens with readily available peptidoglycan‐rich cell walls and penicillin‐binding proteins pertinent to the intended antibacterial action; thus, this targeted group was deemed suitable for determining the preliminary structure–activity relationship of the newly developed compounds [37].

**TABLE 1:** In vitro antibacterial activity with MIC in µg.^a^

Comp	MIC(µg/mL)			
	R	*B. subtilis*	*S. aureus*	*S. epidermidis*
**6**	—	>50	>50	>50
**8a**	H	>50	>50	>50
**8b**	4‐Me	50 ± 1.46	50 ± 1.66	50 ± 1.88
**8c**	3,5‐diMe	25 ± 1.15	25 ± 1.42	50 ± 1.93
**8d**	4‐C_4_H_9_	25 ± 1.07	50 ± 1.54	50 ± 1.63
**8e**	4‐OMe	12.5 ± 0.89	25 ± 1.95	25 ± 1.14
**8f**	3,5‐diOMe	12.5 ± 0.95	12.5 ± 1.02	12.5 ± 1.02
**8g**	4‐Cl	6.25 ± 0.55	6.25 ± 0.98	12.5 ± 1.08
**8h**	4‐F	3.12 ± 0.31	6.25 ± 0.84	6.25 ± 0.86
**8i**	4‐Br	6.25 ± 0.54	12.5 ± 0.88	12.5 ± 0.92
**8j**	3,5‐diCl	1.56 ± 0.22	1.56 ± 0.22	6.25 ± 0.67
**8k**	3,5‐diBr	3.12 ± 0.28	6.25 ± 0.47	6.25 ± 0.82
**8l**	3,5‐diF	1.56 ± 0.18	1.56 ± 0.31	6.25 ± 0.79
**8m**	4‐NO_2_	6.25 ± 0.98	12.5 ± 0.93	25 ± 0.84
**8n**	4‐CN	6.25 ± 0.87	12.5 ± 0.85	12.5 ± 0.92
**Std**	Dicloxacillin	3.12 ± 0.57	6.25 ± 0.62	6.25 ± 0.54

^a^ = Values are mean ± SD of three replicates.

The synthesized compounds demonstrated a wide range of antibacterial activity based on the characteristics of the substituents. Intermediate 6 and compound **8a** exhibited inactivity against all tested strains, with MIC values exceeding 50 µg/mL. Compound **8b** had minimal antibacterial efficacy, with a minimum inhibitory concentration (MIC) of 50 µg/mL against all three bacterial species. Conversely, compounds **8c** and **8d** exhibited modest antibacterial activity, with MIC values between 25 and 50 µg/mL, suggesting that the incorporation of substituted aryl groups on the triazole ring enhances antibacterial efficacy.

The antibacterial activity of the modified compounds was showed to gradually increase. Compounds **8e** and **8f** showed enhanced potency, with MIC values between 12.5 and 25 µg/mL against the evaluated gram‐positive bacterial strains. Enhanced efficacy was noted for **8g**, exhibiting MIC values of 6.25 µg/mL against *B. subtilis* and *S. aureus*, and 12.5 µg/mL against *S. epidermidis*. Compound **8h** exhibited significant antibacterial efficacy, with MIC values of 3.12 µg/mL against *B. subtilis* and 6.25 µg/mL against both *S. aureus* and *S. epidermidis*, equivalent to the reference antibiotic dicloxacillin. Within the synthesized series, compounds **8j** and **8l** were identified as the most effective derivatives, demonstrating MIC values of 1.56 µg/mL against *B. subtilis* and *S. aureus*, and 6.25 µg/mL against *S. epidermidis*. Compounds **8i** and **8k** exhibited significant antibacterial activity, with MIC values between 3.12 and 12.5 µg/mL, while compounds 8m and 8n showed moderate efficacy (MIC = 6.25–25 µg/mL). The biological evaluation indicated that compounds **8h**, **8j**, and **8l** exhibit exceptional antibacterial activity, with inhibitory effects comparable to or surpassing those of dicloxacillin (MIC = 3.12–6.25 µg/mL) against the tested gram‐positive bacterial strains, thus identifying them as the most promising candidates for further study.

Although the present study demonstrates promising antibacterial activity against selected gram‐positive laboratory strains, further investigation against clinically isolated and multidrug‐resistant pathogens, particularly MRSA is warranted to establish the therapeutic relevance and resistance‐profile of the most potent derivatives [38].

### 2.2 Structure–Activity Relationship

The antibacterial efficacy of compounds **8a–8n** demonstrated a distinct structure–activity relationship (SAR), underscoring the substantial impact of the electronic characteristics and substitution pattern of the aryl group on antibacterial strength. Compounds without substituents or possessing weak electron‐donating groups, such as **8a** (H) and **8b** (4‐Me) demonstrated little to weak antibacterial activity (MIC < 50 µg/mL), suggesting that these substituents are inadequate for facilitating efficient interactions with the bacterial target. Likewise, compounds **8c** (3,5‐diMe) and **8d** (4‐C_4_H_9_) exhibited very moderate activity, indicating that augmenting steric bulk via alkyl substitution does not significantly improve antibacterial effectiveness. The incorporation of electron‐donating methoxy groups led to a moderate enhancement in activity, as evidenced by compounds **8e** (4‐OMe) and **8f** (3,5‐diOMe), which demonstrated MIC values ranging from 12.5 to 25 µg/mL. Nonetheless, these compounds exhibited worse potency compared to their halogen‐substituted equivalents. An observable improvement in antibacterial activity occurred with the addition of halogen substituents. Compounds **8g** (4‐Cl) and **8h** (4‐F) exhibited good to exceptional activity, with 8 h showing MIC values akin to those of the reference medication dicloxacillin. The enhanced efficacy of the halogenated derivatives can be ascribed to the electron‐withdrawing characteristics of the halogens, which augment lipophilicity, refine electronic distribution, and fortify hydrophobic and noncovalent interactions with the bacterial target, thus amplifying antibacterial potency.

Among the synthesized derivatives, di‐halogenated compounds had the greatest antibacterial efficacy, highlighting the advantageous influence of numerous electron‐withdrawing substituents on biological activity. Compounds **8j** (3,5‐diCl) and **8l** (3,5‐diF) were identified as the most potent analogues, exhibiting MIC values as low as 1.56 µg/mL against the evaluated Gram‐positive bacterial strains. The results suggest that incorporation of two halogen atoms at the 3,5‐meta positions may contribute to enhanced antibacterial activity through a favorable combination of electronic and structural effects; however, the possible contribution of lipophilicity and hydrophobic interactions remains speculative because these physicochemical properties were not determined in the present study. Compound **8k** (3,5‐diBr) exhibited remarkable antibacterial activity, albeit slightly less powerful than the analogous chloro‐ and fluoro‐substituted derivatives, indicating that both the electronegativity and steric size of the halogen affect biological activity. Conversely, compounds with alternative strong electron‐withdrawing substituents, such as **8m** (4‐NO_2_) and **8n** (4‐CN), demonstrated only moderate antibacterial efficacy. These observations indicate that although electron‐withdrawing substituents typically enhance antibacterial activity, the substitution pattern and the physicochemical properties of the substituent are also vital, with 3,5‐dihalogen substitution yielding the most advantageous balance for effective antibacterial activity.

The enhanced antibacterial activity of 3,5‐diF (**8l**) and 3,5‐diCl (**8j**) can be attributed to the combined electron‐withdrawing effects and favorable steric arrangement of the two halogen substituents. Their symmetric 3,5‐substitution may optimize the aryl moiety for interactions within the penicillin‐binding protein (PBP) binding region. Docking studies showed favorable binding energies of −8.73 kcal/mol (**8l**) and −8.69 kcal/mol (**8j**), with hydrogen‐bond interactions involving THR600, ALA642, GLU602, and ASN464. These interactions may stabilize ligand–PBP complexes and potentially interfere with PBP‐mediated peptidoglycan cross‐linking, thereby contributing to the observed potent antibacterial activity.

The enhanced antibacterial activity observed for the halogenated derivatives, particularly the 3,5‐dichloro (**8j**) and 3,5‐difluoro (**8l**) analogues, may be associated with changes in electronic distribution, steric characteristics, and hydrophobic interactions; however, these factors are proposed as plausible SAR hypotheses, as the corresponding physicochemical properties were not experimentally determined in the present study. Although electron‐withdrawing substituents may influence antibacterial activity, the moderate activity of the nitro‐ and cyano‐substituted derivatives indicates that electronic effects alone cannot account for the observed SAR; therefore, substituent position, steric characteristics, polarity, molecular shape, and target interactions may collectively contribute to antibacterial potency. The findings underscore the essential influence of the electronic characteristics and substitution pattern of the aryl moiety in regulating antibacterial effectiveness and offer significant insights for the systematic design of more potent antibacterial agents derived from the fused imidazo[1,2‐b]pyrazole–1,2,4‐oxadiazole–1,2,3‐triazole framework.

Although **8j** and **8l** exhibited promising activity against the tested *S. aureus* strain (MIC = 1.56 µg/mL), clinically established anti‐MRSA agents such as vancomycin, linezolid, daptomycin and ceftaroline provide an important therapeutic benchmark; however, direct potency comparisons should be interpreted cautiously because these reference antibiotics were not evaluated under identical experimental conditions in the present study.

### 2.3 Antibiofilm Activity

Compounds **8h, 8j, 8k,** and **8l** were selected for preliminary antibiofilm evaluation because they represent structurally distinct and highly active halogenated derivatives, enabling assessment of how halogen identity, number, and substitution pattern influence antibiofilm activity. The antibiofilm efficacy of the most effective antibacterial agents (**8h, 8j, 8k,** and **8l**) was assessed against *B. subtilis*, *S. aureus*, and *S. epidermidis*, with findings compared to the reference medication dicloxacillin (Table 2) [21]. Of the evaluated derivatives, compound **8j** demonstrated the most potent antibiofilm activity, exhibiting the lowest biofilm inhibitory concentration (**BFIC**) values against all three bacterial strains. Compound **8l** exhibited remarkable antibiofilm activity, with BFIC values of 3.59 ± 0.38, 4.38 ± 0.51, and 10.67 ± 1.05 µg/mL against *B. subtilis*, *S. aureus*, and *S. epidermidis*, respectively, equivalent to dicloxacillin (BFIC = 3.88–6.18 µg/mL). Compound 8k demonstrated moderate to strong antibiofilm efficacy, with BFIC values between 5.96 and 11.83 µg/mL, while **8h** showed relatively less inhibitory activity (BFIC = 11.87–18.34 µg/mL). Compounds **8j** and **8l** exhibited the most significant antibiofilm effects, especially against *B. subtilis* and *S. aureus*, highlighting their potential as antibacterial agents that can inhibit biofilm formation and the persistence of gram‐positive bacterial infections.

**TABLE 2:** Biofilm activity of potent compounds.

	Biofilm (µg/mL)		
Compound	*B. subtilis*	*S. aureus*	*S. epidermidis*
**8h**	11.87 ± 0.97^**^	12.38 ± 0.85^**^	18.34 ± 0.82
**8j**	3.19 ± 0.34^***^	5.85 ± 0.79^***^	9.21 ± 0.69^**^
**8k**	5.96 ± 0.65^**^	9.69 ± 0.67^**^	11.83 ± 0.91^**^
**8l**	3.59 ± 0.38^***^	4.38 ± 0.51^***^	10.67 ± 1.05^**^
**Dicloxacillin**	3.88 ± 0.31	4.12 ± 0.29	6.18 ± 0.74

** *P* < 0.05.

*** *P* < 0.001

Compounds **8j** and **8l** exhibited promising inhibition of biofilm formation; however, their effects on mature biofilm architecture, extracellular polymeric substances, and biofilm‐associated virulence require further experimental investigation.

### 2.4 Molecular Docking Studies

In order to obtain molecular‐level understanding of the observed antibacterial efficacy, in silico molecular docking analyses were conducted for compounds **8h, 8j, 8k,** and **8l** against the *S. aureus* (MRSA) penicillin‐binding protein (PDB ID: **1MWT**), utilising penicillin G as the co‐crystallized ligand and dicloxacillin as the reference compound [21]. The docking results (Table 3) demonstrated that all selected compounds displayed advantageous binding affinities for the active region of the target protein (Figures 2, 3, 4, 5). Compound **8k** had the highest binding affinity, with a binding energy of −9.10 kcal/mol, succeeded by **8l** (−8.73 kcal/mol), **8j** (−8.69 kcal/mol), and **8h** (−8.26 kcal/mol). Figure 6 illustrates that these binding energies were equivalent to or superior to those of the reference substances dicloxacillin (−8.94 kcal/mol) and penicillin G (−7.41 kcal/mol). The robust binding affinities demonstrated by the synthesised hybrids align with their encouraging antibacterial properties, reinforcing their promise as effective inhibitors of penicillin‐binding proteins in Gram‐positive bacteria. Multiple stabilizing interactions, such as hydrogen bonding with SER462 and GLN521, halogen interaction with GLU460, and π‐π stacking with ARG445 and TYR446, are responsible for the increased binding of compound 8k. Likewise, 8j and 8l showed robust binding because of important hydrogen bonds with THR600 and neighboring residues, in addition to π‐cation and hydrophobic interactions. Through several hydrogen bonds and π‐interactions, Compound **8h** also demonstrated high affinity. Compared to Penicillin G, Dicloxacillin showed stronger binding through hydrogen bonds and π‐sulfur interactions. The docking results indicate that compounds especially **8k** have good binding efficiency and could be effective against bacteria.

**TABLE 3:** Binding affinity outcomes of compounds from the docking assessment (PDB id‐**1MWT**).

Compounds	B. E (kcal.mol^−1^)	No. of H‐bonds	H‐bond Interactions
**8h**	−8.26	4	THR600, ALA642 & SER643.
**8j**	−8.69	4	THR600, GLU602 & ASN464
**8k**	−9.10	2	SERN462 and GLN521
**8l**	−8.73	3	ALA642, THR600
**Dicloxacillin**	−8.94	4	THR600, SER462 & LYS597.
**Penicillin G**	−7.41	4	SER462, LYS597 & THR600.

**FIGURE 2:**
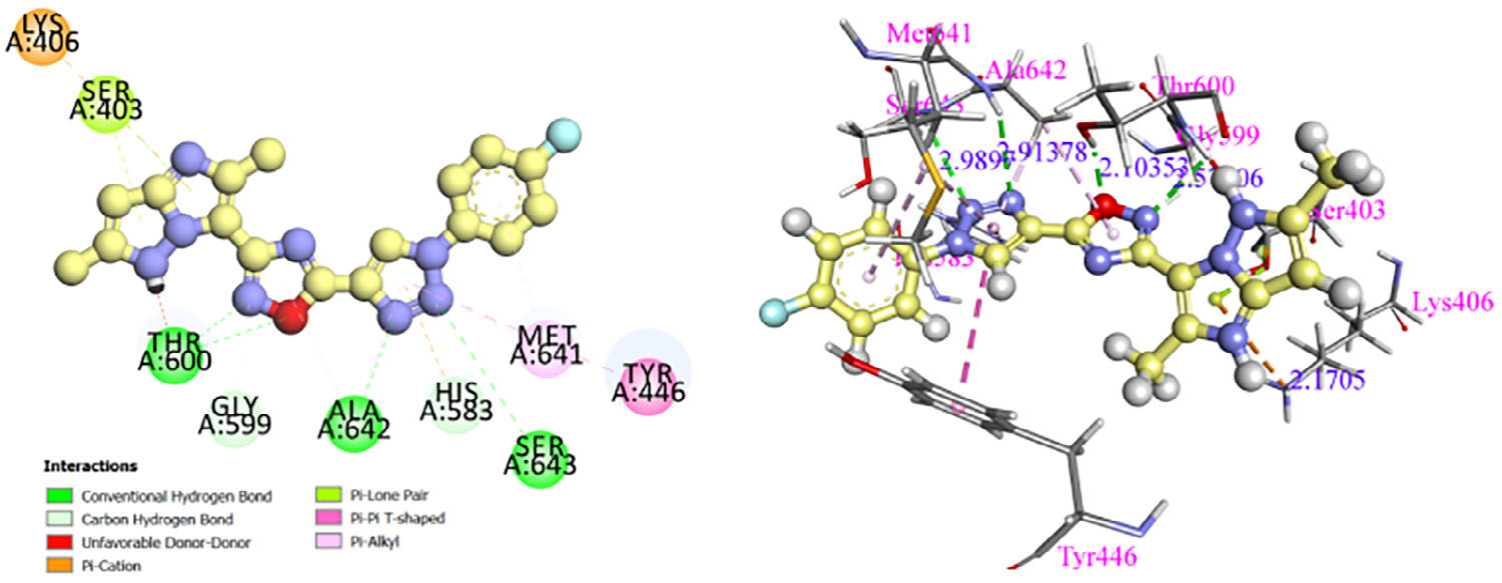
2D and 3D interaction of compound **8h** with penicillin‐binding protein.

**FIGURE 3:**
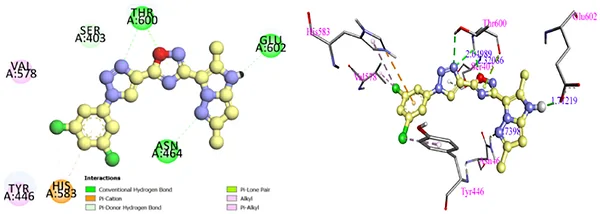
2D and 3D interaction of compound **8j** with penicillin‐binding protein.

**FIGURE 4:**
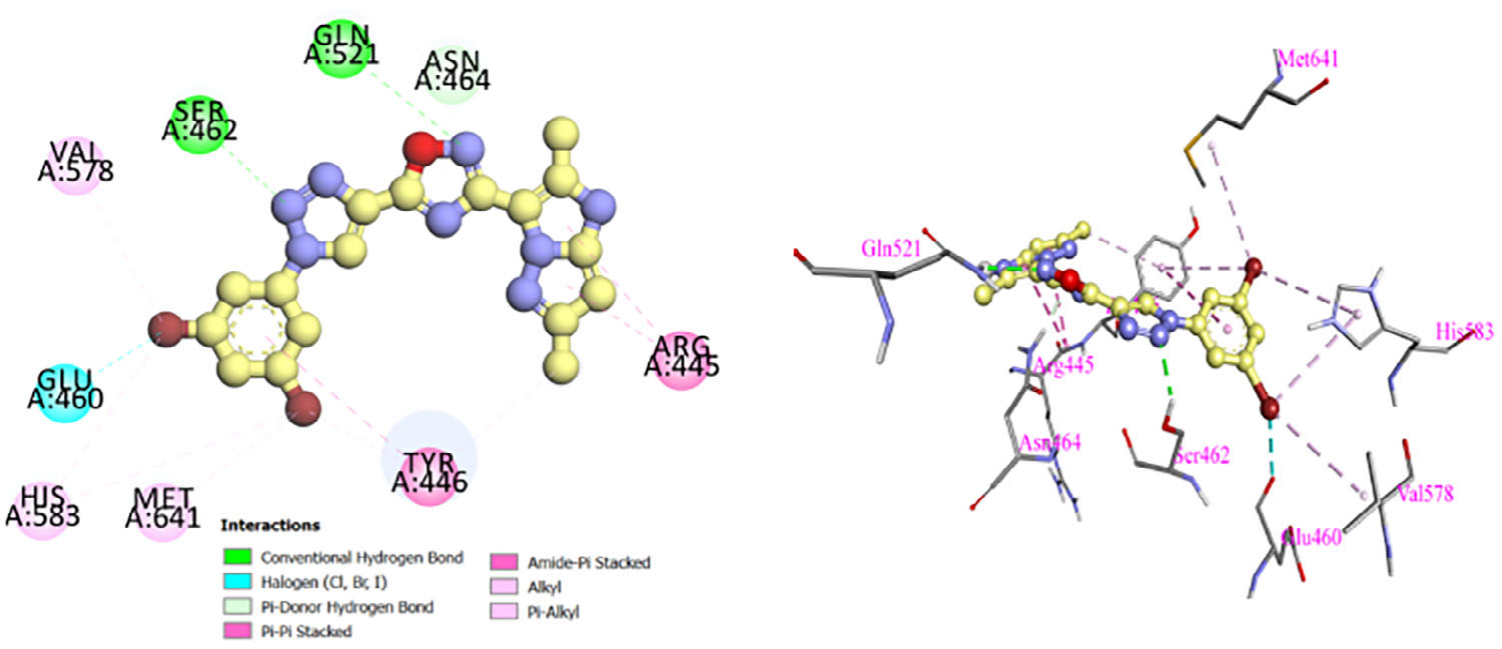
2D and 3D interaction of compound **8k** with penicillin‐binding protein.

**FIGURE 5:**
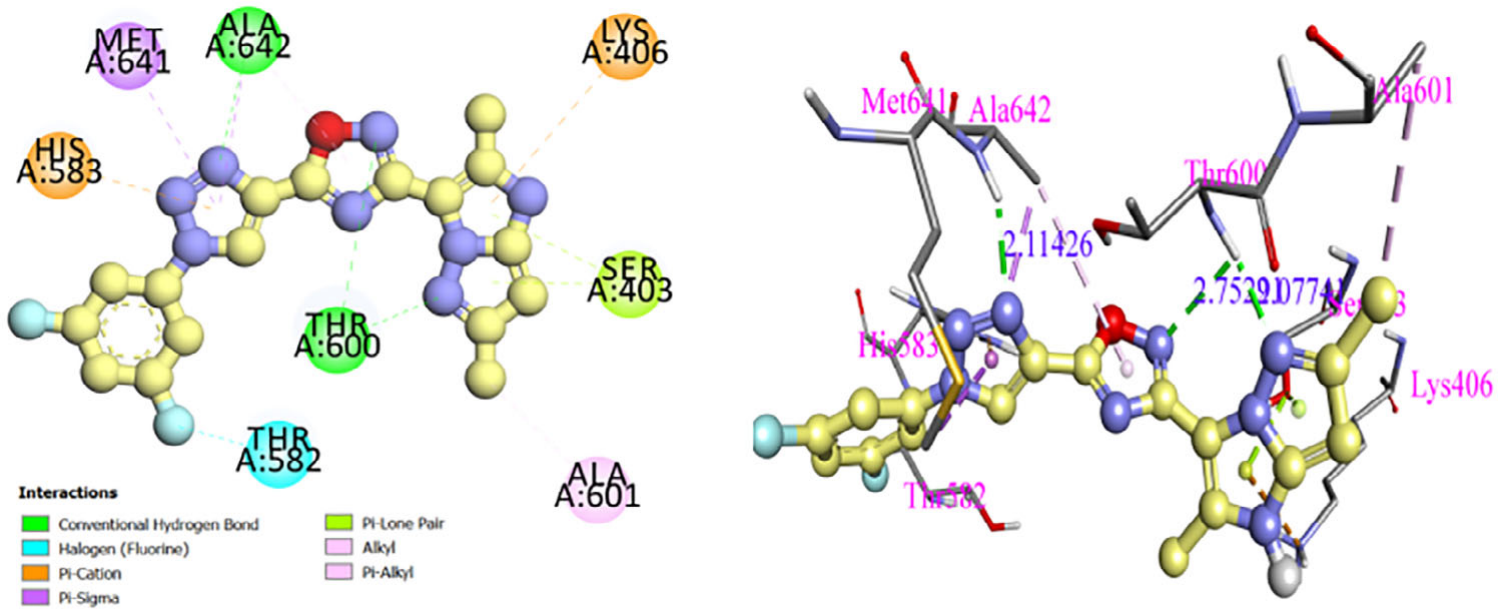
2D and 3D interaction of compound **8l** with penicillin‐binding protein.

**FIGURE 6:**
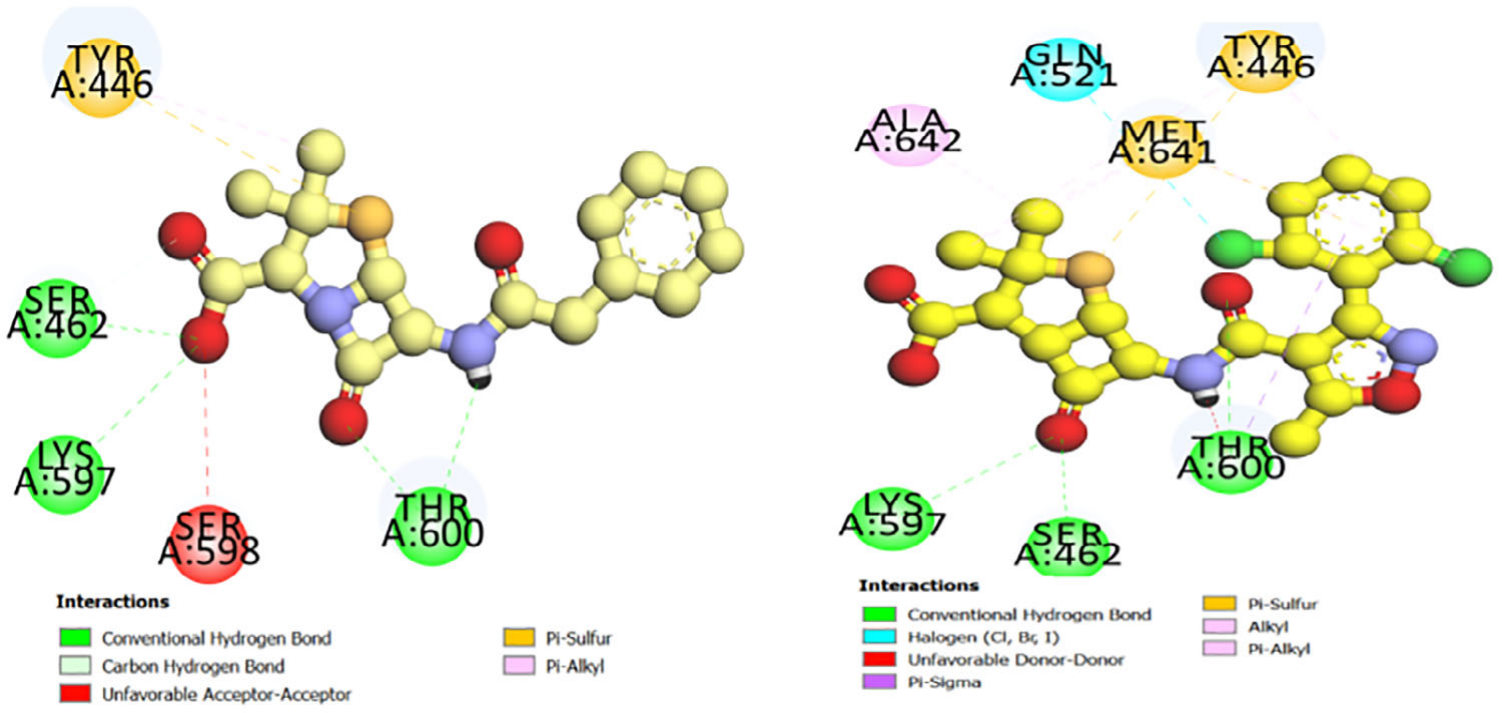
2D interactions of Penicillin G and Dicloxacillin with penicillin‐binding protein.

Molecular docking suggests that the synthesized compounds may interact favorably with the PBP binding site; however, these computational findings do not establish PBP as the definitive biological target, and further biochemical and cellular studies are required for target validation.

The docking–activity discrepancy indicates that PBP binding affinity alone does not determine antibacterial potency. Although **8k** showed the best docking score, **8j** and **8l** exhibited stronger in vitro activity, possibly due to differences in solubility, permeability, cellular uptake, and other physicochemical properties. Thus, docking provides supportive rather than definitive evidence of antibacterial activity. The higher docking affinity of 8k compared with **8j** and **8l** does not directly translate into greater antibacterial potency, suggesting that factors such as solubility, permeability, cellular uptake, and other physicochemical and biological properties may contribute substantially to the observed in vitro activity. Interestingly, although **8k** (3,5‐diBr) exhibited the most favorable docking score (−9.10 kcal/mol), **8j** (3,5‐diCl), and **8l** (3,5‐diF) demonstrated superior antibacterial activity. This discrepancy suggests that target‐binding affinity alone does not determine antibacterial potency. The larger bromine substituents in **8k** may favor interactions within the modeled binding site but could also influence molecular size, solubility, permeability, and cellular uptake. In contrast, the smaller F and Cl substituents in **8l** and **8j** may provide a more favorable overall balance of physicochemical and biological properties. These factors remain plausible explanations and require further experimental validation. The favorable docking score of **8k** suggests a potential interaction with the PBP binding site and provides supportive computational evidence; however, it does not establish antibacterial efficacy or confirm PBP as the definitive biological target.

The docking protocol was further validated by redocking the native co‐crystallized penicillin G ligand into the binding site. The redocked ligand showed good agreement with the experimentally observed co‐crystallized pose, with an RMSD of 1.13 Å (Figure 7) supporting the reliability of the docking protocol used in this study.

**FIGURE 7:**
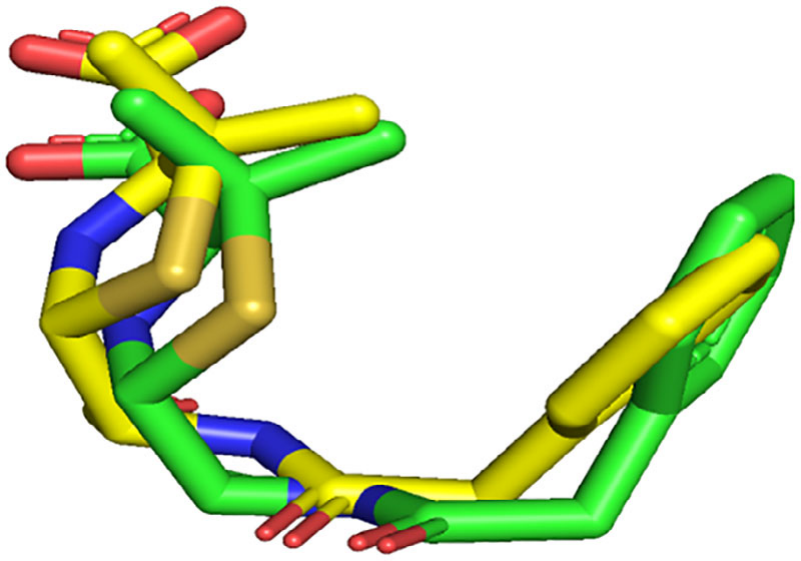
Redocking validation of penicillin G showing the redocked ligand (Green) and co‐crystallized ligand (Yellow), with an RMSD of **1.13 Å**.

### 2.5 Comparison Between In Vitro and In Silico Antibacterial Activity

The results of the in vitro antibacterial activity and the in silico molecular docking was compared, and the comparison revealed that there was a strong overall connection between the findings of the experiments and the findings of the experiments. In terms of antibacterial activity, compounds **8j** and **8l** demonstrated the highest level of effectiveness, with MIC values of 1.56 µg/mL against *B. subtilis* and *S. aureus*. These compounds outperformed the reference medication dicloxacillin. In line with these data, it was showed that both compounds had favourable binding affinities towards the penicillin‐binding protein (PDB ID: 1MWT). The binding energies of these compounds were −8.69 and 8.73 kcal/mol, respectively, which indicates that they have persistent interactions with the ligand. Despite the fact that compound 8k displayed the greatest docking score (−9.10 kcal/mol), its antibacterial activity in vitro was slightly lower than that of compounds **8j** and **8l**. Molecular docking is a useful tool for predicting ligand–protein interactions; however, it does not take into account all of the important biological factors that can influence experimental activity. These factors include membrane permeability, solubility, cellular uptake, and metabolic stability. This discrepancy highlights the fact that molecular docking does not fully account for these factors. In general, the docking results are in agreement with the data obtained in vitro and lend credence to the possibility that compounds **8j, 8k,** and **8l** could be among the most promising antibacterial lead molecules. In silico molecular docking is a powerful technique for guiding lead optimisation, rationalising structure–activity relationships, and prioritizing compounds for biological evaluation, as demonstrated by these data, which further highlight those benefits.

## 3 Experimental Section

Synthesis of 2,5‐dimethyl‐5H‐imidazo[1,2‐b]pyrazole‐3‐carbonitrile (**3**): A stirred solution of 1‐methyl‐1H‐pyrazol‐3‐amine (1) (5.0 g, 0.05 mol) in H_2_O (50 mL) was treated with 3‐oxobutanenitrile (2) (0.055 mol, 1.1 equivalents), followed by the addition of N‐bromosuccinimide (NBS) (2.0 equivalents) at ambient temperature. The resultant reaction mixture was heated to 80°C and agitated for 24 h. The reaction's progress was assessed using thin‐layer chromatography (TLC), which verified the total consumption of the starting material. Upon completion, the reaction mixture was cooled to ambient temperature and subsequently extracted with 100 mL of ethyl acetate. The organic layer was rinsed with saturated aqueous brine (2 × 50 mL), dried with anhydrous Na_2_SO_4_, filtered, and concentrated under reduced pressure to provide the crude product. The crude residue was refined via silica gel column chromatography with a suitable eluent, yielding 2,5‐dimethyl‐5H‐imidazo[1,2‐b]pyrazole‐3‐carbonitrile (3) as a pure compound. The desired product was eluted with 20% EtOAc/Hexane to afford off white solid 3 (82% yield).^1^H NMR (400 MHz, CDCl_3_) δ 7.68 (d, *J* = 8.0 Hz, 1H), 7.24 (d, *J* = 8.0 Hz, 1H), 3.24 (s, 3H, N─CH_3_), 2.17 (s, 3H, ─CH_3_); EI‐MS m/z 161 [M+H]^+^.

Synthesis of 3‐(2,5‐dimethyl‐5H‐imidazo[1,2‐b]pyrazol‐3‐yl)‐5‐ethynyl‐1,2,4‐oxadiazole (**6**): A stirred solution of compound 3 (2.0 g, 0.0125 mol, 1.0 equiv) in dry dichloromethane (DCM) (50 mL) received additions of hydroxylamine hydrochloride (NH_2_OH·HCl) (1.5 equiv) and triethylamine (TEA) (8 mL). The reaction mixture was agitated at ambient temperature for 7 h to produce the appropriate amidoxime intermediate in situ. Thereafter, Vilsmeier reagent (2.0 equivalents) and propiolic acid (5) (1.3 equivalents) were introduced, and the reaction mixture was agitated for a further 7 h at ambient temperature. The reaction's progress was assessed using thin‐layer chromatography (TLC), which verified the total consumption of the starting material. Upon completion, the reaction mixture was sequentially rinsed with saturated aqueous sodium bicarbonate (30 mL) and brine (30 mL). The organic layer was dehydrated using anhydrous Na_2_SO_4_, filtered, and concentrated under reduced pressure to provide the crude product. The crude residue underwent purification via silica gel column chromatography with a suitable eluent, yielding the required 1,2,4‐oxadiazole intermediate (6) in 68% yield. ^1^H NMR (400 MHz, CDCl_3_) δ 7.69 (d, *J* = 8.0 Hz, 1H), 7.23 (d, *J* = 8.0 Hz, 1H), 3.25 (s, 3H, N─CH_3_), 2.29 (s, 1H, ─CH), 2.15 (s, 3H, ─CH_3_); ESI‐MS: 228 [M+H]^+^.

Synthesis of 3‐(2,5‐dimethyl‐5H‐imidazo[1,2‐b]pyrazol‐3‐yl)‐5‐(1‐(aryl)‐1H‐1,2,3‐triazol‐4‐yl)‐1,2,4‐oxadiazole (**8a‐8n**): A stirred solution of alkyne intermediate 6 (0.30 g, 0.0013 mol) and the corresponding aryl azide (7) (0.0015 mol, 1.15 equivalents) in THF (15 mL) was treated with CuI (10 mol%) at ambient temperature. The reaction mixture was agitated for 8–10 h, and the reaction's progress was assessed using thin‐layer chromatography (TLC). Upon completion, the reaction mixture was transferred to cold water (15 mL), and the product was extracted using ethyl acetate (2 × 20 mL). The amalgamated organic extracts were rinsed with brine, desiccated over anhydrous Na_2_SO_4_, filtered, and concentrated under reduced pressure to get the crude product. The crude residue underwent purification using silica gel column chromatography employing a gradient elution of hexane/ethyl acetate (25% EtOAc), yielding the intended fused imidazo[1,2‐b]pyrazole–1,2,4‐oxadiazole–1,2,3‐triazole derivatives **8a–8n** in good to excellent yields.

3‐(2,5‐dimethyl‐5H‐imidazo[1,2‐b]pyrazol‐3‐yl)‐5‐(1‐phenyl‐1H‐1,2,3‐triazol‐4‐yl)‐1,2,4‐oxadiazole (**8a**): M.p: 143‐145°C; ^1^H‐NMR (400 MHz, DMSO‐*d*_6_) δ 8.12 (s, 1H, tri‐H), 7.73 (d, *J* = 8.0 Hz, 1H), 7.60 (d, *J* = 8.0 Hz, 2H, Ar‐H), 7.41 (d, *J* = 8.0 Hz, 1H), 7.36‐7.31 (m, 3H, Ar‐H), 3.63 (s, 3H, N─CH_3_), 2.31 (s, 3H, ─CH_3_); ^13^C‐NMR (100 MHz, DMSO‐*d*_6_) δ 170.75, 164.37, 160.34, 139.51, 137.47, 130.72, 129.31(2C), 127.15, 125.41, 122.42(2C), 118.65, 110.46, 104.27, 36.63, 18.46; ESI‐MS: 347 [M+H]^+^. Anal. Calcd for C_17_H_14_N_8_O: C, 58.95; H, 4.07; N, 32.35. Found: C, 58.92; H, 4.04; N, 32.33.

3‐(2,5‐dimethyl‐5H‐imidazo[1,2‐b]pyrazol‐3‐yl)‐5‐(1‐(p‐tolyl)‐1H‐1,2,3‐triazol‐4‐yl)‐1,2,4‐oxadiazole (**8b**): M.p: 148‐150°C; ^1^H‐NMR (400 MHz, DMSO‐*d*_6_) δ 8.11 (s, 1H, tri‐H), 7.72 (d, *J* = 8.0 Hz, 1H), 7.59 (d, *J* = 8.0 Hz, 2H, Ar‐H), 7.41 (d, *J* = 8.0 Hz, 1H), 7.31 (d, *J* = 8.0 Hz, 2H, Ar‐H), 3.64 (s, 3H, N─CH_3_), 2.32 (s, 3H, ─CH_3_), 2.23 (s, 3H, Ar─CH_3_); ^13^C‐NMR (100 MHz, DMSO‐*d*_6_) δ 170.25, 164.41, 160.30, 139.59, 137.78, 136.75, 132.37, 129.15(2C), 125.57, 124.21(2C), 118.80, 110.43, 104.52, 36.28, 21.56, 18.73; ESI‐MS: 361 [M+H]^+^. Anal. Calcd for C_18_H_16_N_8_O: C, 59.99; H, 4.48; N, 31.09. Found: C, 59.97; H, 4.46; N, 31.06.

3‐(2,5‐dimethyl‐5H‐imidazo[1,2‐b]pyrazol‐3‐yl)‐5‐(1‐(3,5‐dimethylphenyl)‐1H‐1,2,3‐triazol‐4‐yl)‐1,2,4‐oxadiazole (**8c**): M.p: 159‐161°C; ^1^H‐NMR (400 MHz, DMSO‐*d*_6_) δ8.10 (s, 1H, tri‐H),7.73 (d, *J* = 8.0 Hz, 1H), 7.54 (s, 2H, Ar‐H), 7.41 (d, *J* = 8.0 Hz, 1H), 7.18 (s, 1H, Ar‐H), 3.63 (s, 3H, N─CH_3_), 2.32 (s, 3H, ─CH_3_), 2.16 (s, 6H, 2Ar─CH_3_);^13^C‐NMR (100 MHz, DMSO‐*d*_6_) δ 170.88, 164.39, 160.28, 139.72, 138.13(2C), 137.38, 132.40, 126.54, 125.48(2C), 124.53, 118.56, 110.44, 104.11, 36.62, 20.88(2C), 18.16; ESI‐MS: 375 [M+H]^+^. Anal. Calcd for C_19_H_18_N_8_O: C, 60.95; H, 4.85; N, 29.93. Found: C, 60.92; H, 4.82; N, 29.91.

5‐(1‐(4‐butylphenyl)‐1H‐1,2,3‐triazol‐4‐yl)‐3‐(2,5‐dimethyl‐5H‐imidazo[1,2‐b]pyrazol‐3‐yl)‐1,2,4‐oxadiazole (**8d**): M.p: 165‐167°C; ^1^H‐NMR (400 MHz, DMSO‐*d*_6_) δ 8.12 (s, 1H, tri‐H), 7.72 (d, *J* = 8.0 Hz, 1H), 7.60 (d, *J* = 8.0 Hz, 2H, Ar‐H), 7.41 (d, *J* = 8.0 Hz, 1H), 7.32 (d, *J* = 8.0 Hz, 2H, Ar‐H), 3.63 (s, 3H, N─CH_3_), 2.75 (t, *J* = 4.0 Hz, ─CH_2_‐), 2.33 (s, 3H, ─CH_3_), 1.69‐1.63 (m, 2H), 1.36‐1.30 (m, 2H), 0.97 (t, *J* = 4.0 Hz, ─CH_3_); ^13^C‐NMR (100 MHz, DMSO‐*d*_6_) δ 170.35, 164.27, 160.63, 139.70, 138.86, 137.89, 132.38, 128.31(2C), 126.26(2C), 125.25, 118.69, 110.59, 104.48, 36.42, 34.43, 30.24, 21.46, 18.66, 14.31; ESI‐MS: 403 [M+H]^+^. Anal. Calcd for C_21_H_22_N_8_O: C, 62.67; H, 5.51; N, 27.84. Found: C, 62.65; H, 5.48; N, 27.81.

3‐(2,5‐dimethyl‐5H‐imidazo[1,2‐b]pyrazol‐3‐yl)‐5‐(1‐(4‐methoxyphenyl)‐1H‐1,2,3‐triazol‐4‐yl)‐1,2,4‐oxadiazole (**8e**): M.p: 156‐158°C; ^1^H‐NMR (400 MHz, DMSO‐*d*_6_) δ 8.13 (s, 1H, tri‐H),7.73 (d, *J* = 8.0 Hz, 1H), 7.69 (d, *J* = 8.0 Hz, 2H, Ar‐H), 7.42 (d, *J* = 8.0 Hz, 1H), 7.00 (d, *J* = 8.0 Hz, 2H, Ar‐H), 3.86 (s, 3H, O─CH_3_), 3.63 (s, 3H, N─CH_3_), 2.32 (s, 3H, ─CH_3_); ^13^C‐NMR (100 MHz, DMSO‐*d*_6_) δ 170.70, 164.17, 160.55, 159.29, 139.40, 132.57, 131.35, 126.73(2C), 125.49, 118.56, 114.62(2C), 110.48, 104.33, 56.49, 36.62, 18.17; ESI‐MS: 377 [M+H]^+^. Anal. Calcd for C_18_H_16_N_8_O_2_: C, 57.44; H, 4.29; N, 29.77. Found: C, 57.42; H, 4.26; N, 29.75.

5‐(1‐(3,5‐dimethoxyphenyl)‐1H‐1,2,3‐triazol‐4‐yl)‐3‐(2,5‐dimethyl‐5H‐imidazo[1,2‐b]pyrazol‐3‐yl)‐1,2,4‐oxadiazole (**8f**): M.p: 168‐170°C; ^1^H‐NMR (400 MHz, DMSO‐*d*_6_) δ 8.10 (s, 1H, tri‐H), 7.71 (d, *J* = 8.0 Hz, 1H), 7.61 (s, 2H, Ar‐H), 7.40 (d, *J* = 8.0 Hz, 1H), 6.97 (s, 1H), 3.82 (s, 6H, Ar‐OCH_3_), 3.61 (s, 3H, N─CH_3_), 2.31 (s, 3H, ─CH_3_);^13^C‐NMR (100 MHz, DMSO‐*d*_6_) δ 170.29, 164.35, 160.53, 158.40(2C), 140.46, 139.30, 132.57, 125.58, 118.57, 110.40, 104.50, 102.18(2C), 98.60, 55.10(2C), 36.32, 18.45; ESI‐MS: 407 [M+H]^+^. Anal. Calcd for C_19_H_18_N_8_O_3_: C, 56.15; H, 4.46; N, 27.57. Found: C, 56.11; H, 4.43; N, 27.54.

5‐(1‐(4‐chlorophenyl)‐1H‐1,2,3‐triazol‐4‐yl)‐3‐(2,5‐dimethyl‐5H‐imidazo[1,2‐b]pyrazol‐3‐yl)‐1,2,4‐oxadiazole (**8g**): M.p: 181‐183°C; ^1^H‐NMR (400 MHz, DMSO‐*d*_6_) δ 8.13 (s, 1H, tri‐H), 7.78 (d, *J* = 8.0 Hz, 2H, Ar‐H), 7.72 (d, *J* = 8.0 Hz, 1H), 7.46 (d, *J* = 8.0 Hz, 2H, Ar‐H), 7.41 (d, *J* = 8.0 Hz, 1H), 3.63 (s, 3H, N─CH_3_), 2.32 (s, 3H, ─CH_3_); ^13^C‐NMR (100 MHz, DMSO‐*d*_6_) δ 170.56, 164.39, 160.69, 139.22, 136.80, 133.36, 132.64, 129.31(2C), 125.40, 124.18(2C), 118.73, 110.54, 104.46, 36.64, 18.44.; ESI‐MS: 381 [M+H]^+^. Anal. Calcd for C_17_H_13_ClN_8_O: C, 53.62; H, 3.44; N, 29.43. Found: C, 53.59; H, 3.42; N, 29.41.

3‐(2,5‐dimethyl‐5H‐imidazo[1,2‐b]pyrazol‐3‐yl)‐5‐(1‐(4‐fluorophenyl)‐1H‐1,2,3‐triazol‐4‐yl)‐1,2,4‐oxadiazole (**8h**): M.p: 150‐152°C; ^1^H‐NMR (400 MHz, DMSO‐*d*_6_) δ 8.18 (s, 1H, tri‐H), 8.09 (d, *J* = 8.0 Hz, 2H, Ar‐H), 7.81 (d, *J* = 8.0 Hz, 2H, Ar‐H), 7.73 (d, *J* = 8.0 Hz, 1H), 7.42 (d, *J* = 8.0 Hz, 1H), 3.64 (s, 3H, N─CH_3_), 2.33 (s, 3H, ─CH_3_); ^13^C‐NMR (100 MHz, DMSO‐*d*_6_) δ 170.48, 164.57, 161.62, 160.22, 159.56, 139.72, 133.71, 132.33, 125.48, 123.41, 123.35, 118.83, 117.84, 117.63, 110.76, 104.58, 36.35, 18.46; ESI‐MS: 365 [M+H]^+^. Anal. Calcd for C_17_H_13_FN_8_O: C, 56.04; H, 3.60; N, 30.76. Found: C, 56.01; H, 3.57; N, 30.78.

5‐(1‐(4‐bromophenyl)‐1H‐1,2,3‐triazol‐4‐yl)‐3‐(2,5‐dimethyl‐5H‐imidazo[1,2‐b]pyrazol‐3‐yl)‐1,2,4‐oxadiazole (**8i**): M.p: 145‐147°C; ^1^H‐NMR (400 MHz, DMSO‐*d*_6_) δ 8.11 (s, 1H, tri‐H),7.73 (d, *J* = 8.0 Hz, 1H), 7.62 (d, *J* = 8.0 Hz, 2H, Ar‐H), 7.54 (d, *J* = 8.0 Hz, 2H, Ar‐H), 7.41 (d, *J* = 8.0 Hz, 1H), 3.63 (s, 3H, N─CH_3_), 2.33 (s, 3H, ─CH_3_); ^13^C‐NMR (100 MHz, DMSO‐*d*_6_) δ 170.68, 164.32, 160.43, 139.35, 135.11, 132.23, 131.29(2C), 125.40, 123.69(2C), 120.58, 118.17, 110.82, 104.24, 36.31, 18.44; ESI‐MS: 425 [M+H]^+^. Anal. Calcd for C_17_H_13_BrN_8_O: C, 48.02; H, 3.08; N, 26.35. Found: C, 48.00; H, 3.05; N, 26.38.

5‐(1‐(3,5‐dichlorophenyl)‐1H‐1,2,3‐triazol‐4‐yl)‐3‐(2,5‐dimethyl‐5H‐imidazo[1,2‐b]pyrazol‐3‐yl)‐1,2,4‐oxadiazole (**8j**): M.p: 161‐163°C; ^1^H‐NMR (400 MHz, DMSO‐*d*_6_) δ 8.12 (s, 1H, tri‐H),7.74 (d, *J* = 8.0 Hz, 1H), 7.66 (s, 2H, Ar‐H), 7.42 (d, *J* = 8.0 Hz, 1H), 7.30 (s, 1H, Ar‐H), 3.64 (s, 3H, N─CH_3_), 2.33 (s, 3H, ─CH_3_); ^13^C‐NMR (100 MHz, DMSO‐*d*_6_) δ_C_ 170.51, 164.56, 160.58, 139.17, 138.15, 135.70(2C), 132.63, 125.38, 124.12, 122.45(2C), 118.79, 110.63, 104.35, 36.32, 18.45; ESI‐MS: 415 [M+H]^+^. Anal. Calcd for C_17_H_12_Cl_2_N_8_O: C, 49.17; H, 2.91; N, 26.99. Found: C, 49.15; H, 2.88; N, 26.95.

5‐(1‐(3,5‐dibromophenyl)‐1H‐1,2,3‐triazol‐4‐yl)‐3‐(2,5‐dimethyl‐5H‐imidazo[1,2‐b]pyrazol‐3‐yl)‐1,2,4‐oxadiazole (**8k**): M.p: 160‐162°C; ^1^H‐NMR (400 MHz, DMSO‐*d*_6_) δ8.14 (s, 1H, tri‐H), 7.74 (d, *J* = 8.0 Hz, 1H), 7.58 (s, 2H, Ar‐H), 7.41 (d, *J* = 8.0 Hz, 1H), 7.32 (s, 1H, Ar‐H), 3.64 (s, 3H, N─CH_3_), 2.33 (s, 3H, ─CH_3_);^13^C‐NMR (100 MHz, DMSO‐*d*_6_) δ 170.34, 164.54, 160.49, 139.50, 137.70, 132.58, 129.56, 127.73(2C), 125.44, 121.54(2C), 118.71, 110.52, 104.45, 36.65, 18.19; ESI‐MS: 503 [M+H]^+^. Anal. Calcd for C_17_H_12_Br_2_N_8_O: C, 40.50; H, 2.40; N, 22.23. Found: C, 40.48; H, 2.37; N, 22.20.

5‐(1‐(3,5‐difluorophenyl)‐1H‐1,2,3‐triazol‐4‐yl)‐3‐(2,5‐dimethyl‐5H‐imidazo[1,2‐b]pyrazol‐3‐yl)‐1,2,4‐oxadiazole (**8l**): M.p: 169‐171°C; ^1^H‐NMR (400 MHz, DMSO‐*d*_6_) δ 8.15 (s, 1H, tri‐H), 8.08 (s, 2H, Ar‐H), 7.84 (s, 1H, Ar‐H), 7.73 (d, *J* = 8.0 Hz, 1H), 7.42 (d, *J* = 8.0 Hz, 1H), 3.64 (s, 3H, N─CH_3_), 2.32 (s, 3H, ─CH_3_); ^13^C‐NMR (100 MHz, DMSO‐*d*_6_) δ 170.45, 164.37, 163.27, 163.20, 161.18, 161.11, 160.13, 139.43, 135.20, 135.13, 135.05, 132.34, 125.59, 118.64, 110.60, 106.55, 106.34, 106.14, 104.29, 103.46, 103.40, 103.19, 103.12, 36.61, 18.76; ESI‐MS: 383 [M+H]^+^. Anal. Calcd for C_17_H_12_F_2_N_8_O: C, 53.41; H, 3.16; N, 29.31. Found: C, 53.38; H, 3.12; N, 29.29.

3‐(2,5‐dimethyl‐5H‐imidazo[1,2‐b]pyrazol‐3‐yl)‐5‐(1‐(4‐nitrophenyl)‐1H‐1,2,3‐triazol‐4‐yl)‐1,2,4‐oxadiazole (**8m**): M.p: 176‐178°C; ^1^H‐NMR (400 MHz, DMSO‐*d*_6_) δ 8.28 (d, *J* = 8.0 Hz, 2H, Ar‐H), 8.15 (s, 1H, tri‐H), 8.06(d, *J* = 8.0 Hz, 2H, Ar‐H), 7.75 (d, *J* = 8.0 Hz, 1H), 7.43 (d, *J* = 8.0 Hz, 1H), 3.64 (s, 3H, N─CH_3_), 2.33 (s, 3H, ─CH_3_); ^13^C‐NMR (100 MHz, DMSO‐*d*_6_) δ 170.26, 164.49, 160.48, 146.33, 140.65, 139.45, 132.81, 126.78(2C), 125.28, 123.62(2C), 118.52, 110.67, 104.36, 36.29, 18.76; ESI‐MS: 392 [M+H]^+^. Anal. Calcd for C_17_H_13_N_9_O_3_: C, 52.17; H, 3.35; N, 32.21. Found: C, 52.15; H, 3.33; N, 32.25.

4‐(4‐(3‐(2,5‐dimethyl‐5H‐imidazo[1,2‐b]pyrazol‐3‐yl)‐1,2,4‐oxadiazol‐5‐yl)‐1H‐1,2,3‐triazol‐1‐yl)benzonitrile (**8n**): M.p: 181‐183°C; ^1^H‐NMR (400 MHz, DMSO‐*d*_6_) δ 8.13 (s, 1H, tri‐H), 7.86 (d, *J* = 8.0 Hz, 2H, Ar‐H), 7.73 (d, *J* = 8.0 Hz, 1H), 7.51 (d, *J* = 8.0 Hz, 2H, Ar‐H), 7.40 (d, *J* = 8.0 Hz, 1H), 3.63 (s, 3H, N─CH_3_), 2.33 (s, 3H, ─CH_3_); ^13^C‐NMR (100 MHz, DMSO‐*d*_6_) δ 170.33, 164.53, 160.54, 139.29, 138.27, 132.43, 128.77(2C), 126.64(2C), 125.54, 119.71, 118.56, 116.23, 110.45, 104.29, 36.64, 18.47; ESI‐MS: 372 [M+H]^+^. Anal. Calcd for C_18_H_13_N_9_O: C, 58.22; H, 3.53; N, 33.95. Found: C, 58.19; H, 3.51; N, 33.99.

### 3.1 Bacterial Strains and Culture Conditions

*B. subtilis, S. aureus, and S. epidermidis* strains were used for antibacterial and antibiofilm studies. Bacterial strains were cultured in Mueller–Hinton broth (MHB) at 37°C under aerobic conditions. Fresh overnight cultures were diluted to the required inoculum density before each experiment.

### 3.2 Determination of Minimum Inhibitory Concentration (MIC)

The MIC of the synthesized compounds (**6a–6o**) was determined using the broth microdilution method, following Clinical and Laboratory Standards Institute (CLSI) guidelines. Briefly, serial two‐fold dilutions of the test compounds ranging from 0.78 to 100 µg/mL (All compounds were initially dissolved in 10% DMSO with in well conc less than 0.01% DMSO) were prepared in MHB in sterile 96‐well microtiter plates. Bacterial suspensions were adjusted to approximately 1 × 10^6^ CFU/mL, and 100 µL of the suspension was added to each well containing 100 µL of the compound solution. Dicloxacillin was used as the reference antibiotic control, while wells containing broth and bacteria without compounds served as growth controls. Plates were incubated at 37°C for 18–24 h, and bacterial growth was assessed visually and spectrophotometrically. The MIC was defined as the lowest concentration of compound that completely inhibited visible bacterial growth. All MIC determinations were performed using three independent biological replicates, with independently prepared bacterial inocula and assay preparations. The results are expressed as mean ± standard deviation (SD) of the three independent experiments.

### 3.3 Antibiofilm Activity Assay

The anti‐biofilm activity of selected compounds was evaluated using a crystal violet–based microtiter plate assay as described. Briefly, bacterial cultures were grown overnight and diluted in MHB supplemented with 1% glucose to a final concentration of 1 × 10^6^ CFU/mL. Aliquots (200 µL) were dispensed into sterile 96‐well plates containing test compounds at the desired concentrations. Plates were incubated at 37°C for 24 h to allow biofilm formation. Following incubation, non‐adherent cells were removed by gently washing the wells with phosphate‐buffered saline (PBS). The remaining biofilms were fixed with methanol, stained with 0.1% (w/v) crystal violet for 15 min, and excess stain was removed by washing with distilled water. The bound dye was solubilized using 95% ethanol, and absorbance was measured at 570 nm. Biofilm inhibition was calculated relative to untreated control wells. Dicloxacillin was included as a reference control. All assays were conducted in triplicate.

Values are expressed as mean ± SD of three independent experiments. Statistical significance was determined by [one‐way ANOVA followed by an appropriate post‐hoc test], with comparisons made against the untreated control. *p < 0.05; *p* < 0.001

$$\begin{array}{ccc} & & \text{Biofilm inhibition}\left(\%\right)\\ & & \;=\left[\left(\text{OD control}-\text{OD sample}\right)/\text{OD control}\right]\times 100 \end{array}$$

## 4 Molecular Docking

Molecular docking was performed to investigate the binding interactions of the potent derivatives (**8h, 8j, 8k** and **8l**) with penicillin‐binding protein from methicillin‐resistant *S. aureus* (MRSA), using Penicillin G and Dicloxacillin as reference standards. Two‐dimensional structures of the test compounds and standard drugs were drawn using ChemBioDraw Ultra 12.0 (CambridgeSoft). These were converted into energy‐minimized three‐dimensional conformations using Gaussian 09 at the B3LYP/6‐311G(d,p) level of theory. All ligands were protonated at physiological pH (7.4) and Gasteiger partial charges and rotatable bonds were assigned using AutoDock Tools (ADT) v1.5.6. The crystal structure of MRSA penicillin‐binding protein (PDB ID: 1MWT) was retrieved from the RCSB Protein Data Bank (www.rcsb.org). All co‐crystallized water molecules and the bound ligand were removed; no cofactors were present in the structure. Polar hydrogens were added, and the protein was energy‐minimized and optimized for docking using UCSF Chimera 1.10.1. The receptor was held rigid throughout docking, while ligands were treated with full torsional flexibility. Docking calculations were performed using AutoDock 4.2 in conjunction with ADT v1.5.6 (MGLTools, Scripps Research Institute; http://mgltools.scripps.edu), employing the Lamarckian genetic algorithm with a population size of 150 and a maximum of 2.5 × 10^6^energy evaluations. Binding affinities were estimated using the AutoDock 4.2 empirical free energy scoring function. The grid box was centered on the active site using AutoDock Vina, with the following parameters: center (Å): *x* = 28.56, *y* = 29.385, *z* = 86.904; grid size (Å) = 22 × 18 × = 15; grid spacing = 0.375 Å. The donor–acceptor distance for hydrogen‐bonding interactions was set at 1.9 Å. For each ligand, 10 independent docking runs were performed, generating 10 docked conformations saved in PDBQT format, and the lowest‐energy pose was selected for further analysis. To validate the docking protocol, the co‐crystallized ligand was extracted from **1MWT** and re‐docked into the same active site under identical parameters. The root‐mean‐square deviation (RMSD) between the re‐docked and original crystallographic pose was 1.13 Å, confirming the reliability and reproducibility of the docking protocol. Final docked complexes were analyzed for binding energy, hydrogen bonding and hydrophobic interactions, and visualized in 2D and 3D using Discovery Studio Visualizer 4.1.0.

## 5 Conclusion

A efficient and modular synthetic approach was effectively established for the synthesis of a new series of fused imidazo[1,2‐b]pyrazole–1,2,4‐oxadiazole–1,2,3‐triazole hybrids. The molecular hybridization of these pharmacologically significant heterocyclic frameworks produced drugs exhibiting promising antibacterial and antibiofilm properties against Gram‐positive bacterial strains. Among the synthesized derivatives, compounds **8j** and **8l** had the highest antibacterial efficacy, displaying MIC values equivalent to or superior to those of the reference antibiotic dicloxacillin. Moreover, compound 8k exhibited the greatest binding affinity for the penicillin‐binding protein in molecular docking analyses, corroborating its robust interaction with the target enzyme. SAR research demonstrated that electron‐withdrawing substituents, especially 3,5‐dihalogen groups, significantly enhance antibacterial and antibiofilm actions. A strong correlation between the in vitro biological assessment and in silico molecular docking findings further substantiates the efficacy of these hybrid compounds as inhibitors of gram‐positive bacterial pathogens. The fused imidazo[1,2‐b]pyrazole–1,2,4‐oxadiazole–1,2,3‐triazole hybrids are attractive lead molecules for the development of next‐generation antibacterial treatments targeting resistant bacterial infections. The docking results provide supportive in silico evidence for possible PBP interactions, while the precise antibacterial mechanism and biological target of these compounds remain to be experimentally validated. Further studies, including cytotoxicity, resistant bacterial isolates, target‐validation, mechanistic, pharmacological, and in vivo investigations, are required to establish their selectivity, mechanism of action, and therapeutic potential.

## Author Contributions

**S. Kavitha** involved designing the work and responsible conceptualization, coordination and overall supervision of the entire work presented in the submitted manuscript. **Suresh Kumar Murahari** was involved in the synthesis, separation, purification and characterization of all the target compounds presented in the manuscript and molecular docking studies.

## Conflicts of Interest

The authors declare no conflicts of interest.

## Supporting information



Experimental procedures, ^1^H‐NMR, ^13^C‐NMR, and Mass copies (Figures S1–S31) available in supporting information.
**Supporting File 1**: cbdv71816‐sup‐0001‐SuppMat.docx

## Data Availability

Data are available from the authors upon request.
