# Supplementary material for: Imidazo[1,2‐b]Pyrazole‐1,2,4‐oxadiazole Linked 1,2,3‐Triazoles: Synthesis, Antibacterial, Biofilm, and In Silico Studies
Source: Chem Biodivers. 2026 Oct 12;23(10):e71816. doi: 10.1002/cbdv.71816 (PMC13659034; doi:10.1002/cbdv.71816)
Supplement: Supplementary file 1 [file CBDV-23-e71816-s001.docx]

**Supporting information**

**Imidazo[1,2-b]pyrazole-1,2,4-oxadiazole linked 1,2,3-triazoles: Synthesis, antibacterial, biofilm and in silico studies**

SURESH KUMAR MURAHARI, KAVITHA SIDDOJU*

*Department of Chemistry, Chaitanya (Deemed to be University), Hyderabad, India*

*E-mail: [kavithavbr@gmail.com](mailto:kavithavbr@gmail.com)

**Experimental section**

All commercially available chemicals were subsequently employed without purification. All chemicals were evaluated for purity utilizing Merck 60F254 silica gel thin-layer chromatography plates. Melting points were documented uncorrected utilizing hot-stage melting point apparatus from Ernst Leitz Wetzlar, Germany. The ^1^H and ^13^C NMR spectra were obtained using the Mercuryplus spectrometer, functioning at 400 MHz for ^1^H and 100 MHz for ^13^C. The ^1^H NMR spectra were referenced to Me_4_Si and residual DMSO, while the ^13^C NMR was referenced to DMSO. The ESI (electrospray ionisation) mass spectra were acquired at an ionising voltage of 70 eV using a Shimadzu QP5050A quadrupole mass spectrometer. Elemental analyses were conducted on a Perkin-Elmer 240 C Elemental Analyser.

**Synthesis of 2,5-dimethyl-5H-imidazo[1,2-b]pyrazole-3-carbonitrile (3):** To a stirred solution of 1-Methyl-1H-pyrazol-3-amine (1) (5g, 0.05mol) in H_2_O (50 mL) was added 3-oxobutanenitrile (2) (0.055mol 1.1 eq), followed by NBS (2.00 eq) at room temperature. The resultant reaction mixture was stirred at 80° C for 24 h, progress of the reaction was monitored by TLC, and from TLC starting materials was consumed. Reaction mixture was cooled to room temperature and diluted with ethyl acetate (100 mL), washed the organic layer with sat. aq brine solution (2x50 ml), dried over Na_2_SO_4_, filtered and concentrated under reduced pressure to obtained crude. The crude was purified by column chromatography by using silica gel. The desired product was eluted with 20% EtOAc/Hexane to afford off white solid **3** (82% yield).^1^H NMR (400 MHz, CDCl_3_) δ 7.68 (d, *J* = 8.0 Hz, 1H), 7.24 (d, *J* = 8.0 Hz, 1H), 3.24 (s, 3H, N-CH_3_), 2.17 (s, 3H, -CH_3_);EI-MS m/z 161 [M+H].

**Synthesis of 3-(2,5-dimethyl-5H-imidazo[1,2-b]pyrazol-3-yl)-5-ethynyl-1,2,4-oxadiazole (6):** To 50 mL of dry DCM, add 2g (0.0125 mol, 1 eq) of **3**, 1.5 eq of NH₂OH.HCl, and 8 mL of triethylamine. Stir at room temperature for 7 hours. After adding the Vilsmeier reagent (2.0 eq) and propiolic acid (**5**) (1.3 eq), it was stirred for a further seven hours at the same temperature. The reaction was complete, according to TLC. Thereafter, 30 ml of brine and 30 mL of saturated NaHCO_3_ were used to wash the reaction mixture. To get the crude product, the organic layer was dried using Na_2_SO_4_, filtered, and the solvent removed. Using silica gel and column chromatography, the crude was refined. The desired product was eluted with 25% EtOAc/Hexane to afford off White solid **6** (68% yield). ^1^H NMR (400 MHz, CDCl_3_) δ 7.69 (d, *J* = 8.0 Hz, 1H), 7.23 (d, *J* = 8.0 Hz, 1H), 3.25 (s, 3H, N-CH_3_), 2.29 (s, 1H, -CH), 2.15 (s, 3H, -CH_3_);ESI-MS: 228 [M+H]^+^.

**Synthesis of 3-(2,5-dimethyl-5H-imidazo[1,2-b]pyrazol-3-yl)-5-(1-(aryl)-1H-1,2,3-triazol-4-yl)-1,2,4-oxadiazole (8a-8n):** A solution of alkyne (**6**) (0.3g 0.0013 mol) and aryl azide (**7**) (0.0015 mmol) in THF (15 mL) was mixed with CuI (10 mol%). The resulting mixture was agitated at room temperature for 8-10 hours. Following the completion of the reaction, the reaction mixture was mixed with cold water (15 mL), and the resulting product was extracted using ethyl acetate (2 × 20 mL). The organic layer, which comprised a combination of substances, was cleansed with brine and dehydrated using anhydrous Na_2_SO_4_. Following filtration, the solvent was removed under reduced pressure and the impure product obtained was subjected to column chromatography using a gradient of hexane and ethyl acetate (25% EtOAc). This process resulted in the isolation of compounds **8a-8n** with high yields ranging from good to excellent.

**3-(2,5-dimethyl-5H-imidazo[1,2-b]pyrazol-3-yl)-5-(1-phenyl-1H-1,2,3-triazol-4-yl)-1,2,4-oxadiazole (8a):** M.p: 143-145 ^o^C; ^1^H-NMR (400 MHz, DMSO-*d*_6_) δ 8.12 (s, 1H, tri-H), 7.73 (d, *J* = 8.0 Hz, 1H), 7.60 (d, *J* = 8.0 Hz, 2H, Ar-H), 7.41 (d, *J* = 8.0 Hz, 1H), 7.36-7.31 (m, 3H, Ar-H), 3.63 (s, 3H, N-CH_3_), 2.31 (s, 3H, -CH_3_); ^13^C-NMR (100 MHz, DMSO-*d*_6_) δ 170.75, 164.37, 160.34, 139.51, 137.47, 130.72, 129.31(2C), 127.15, 125.41, 122.42(2C), 118.65, 110.46, 104.27, 36.63, 18.46; ESI-MS: 347 [M+H]^+^. Anal. Calcd for C_17_H_14_N_8_O: C, 58.95; H, 4.07; N, 32.35. Found: C, 58.92; H, 4.04; N, 32.33.

**3-(2,5-dimethyl-5H-imidazo[1,2-b]pyrazol-3-yl)-5-(1-(p-tolyl)-1H-1,2,3-triazol-4-yl)-1,2,4-oxadiazole (8b):** M.p: 148-150 ^o^C; ^1^H-NMR (400 MHz, DMSO-*d*_6_) δ 8.11 (s, 1H, tri-H), 7.72 (d, *J* = 8.0 Hz, 1H), 7.59 (d, *J* = 8.0 Hz, 2H, Ar-H), 7.41 (d, *J* = 8.0 Hz, 1H), 7.31 (d, *J* = 8.0 Hz, 2H, Ar-H), 3.64 (s, 3H, N-CH_3_), 2.32 (s, 3H, -CH_3_), 2.23 (s, 3H, Ar-CH_3_); ^13^C-NMR (100 MHz, DMSO-*d*_6_) δ 170.25, 164.41, 160.30, 139.59, 137.78, 136.75, 132.37, 129.15(2C), 125.57, 124.21(2C), 118.80, 110.43, 104.52, 36.28, 21.56, 18.73; ESI-MS: 361 [M+H]^+^. Anal. Calcd for C_18_H_16_N_8_O: C, 59.99; H, 4.48; N, 31.09. Found: C, 59.97; H, 4.46; N, 31.06.

**3-(2,5-dimethyl-5H-imidazo[1,2-b]pyrazol-3-yl)-5-(1-(3,5-dimethylphenyl)-1H-1,2,3-triazol-4-yl)-1,2,4-oxadiazole (8c):** M.p: 159-161 ^o^C; ^1^H-NMR (400 MHz, DMSO-*d*_6_) δ8.10 (s, 1H, tri-H),7.73 (d, *J* = 8.0 Hz, 1H), 7.54 (s, 2H, Ar-H), 7.41 (d, *J* = 8.0 Hz, 1H), 7.18 (s, 1H, Ar-H), 3.63 (s, 3H, N-CH_3_), 2.32 (s, 3H, -CH_3_), 2.16 (s, 6H, 2Ar-CH_3_);^13^C-NMR (100 MHz, DMSO-*d*_6_) δ 170.88, 164.39, 160.28, 139.72, 138.13(2C), 137.38, 132.40, 126.54, 125.48(2C), 124.53, 118.56, 110.44, 104.11, 36.62, 20.88(2C), 18.16; ESI-MS: 375 [M+H]^+^. Anal. Calcd for C_19_H_18_N_8_O: C, 60.95; H, 4.85; N, 29.93. Found: C, 60.92; H, 4.82; N, 29.91.

**5-(1-(4-butylphenyl)-1H-1,2,3-triazol-4-yl)-3-(2,5-dimethyl-5H-imidazo[1,2-b]pyrazol-3-yl)-1,2,4-oxadiazole (8d):** M.p: 165-167 ^o^C; ^1^H-NMR (400 MHz, DMSO-*d*_6_) δ 8.12 (s, 1H, tri-H), 7.72 (d, *J* = 8.0 Hz, 1H), 7.60 (d, *J* = 8.0 Hz, 2H, Ar-H), 7.41 (d, *J* = 8.0 Hz, 1H), 7.32 (d, *J* = 8.0 Hz, 2H, Ar-H), 3.63 (s, 3H, N-CH_3_), 2.75 (t, *J* = 4.0 Hz, -CH_2_-), 2.33 (s, 3H, -CH_3_), 1.69-1.63 (m, 2H), 1.36-1.30 (m, 2H), 0.97 (t, *J* = 4.0 Hz, -CH_3_); ^13^C-NMR (100 MHz, DMSO-*d*_6_) δ 170.35, 164.27, 160.63, 139.70, 138.86, 137.89, 132.38, 128.31(2C), 126.26(2C), 125.25, 118.69, 110.59, 104.48, 36.42, 34.43, 30.24, 21.46, 18.66, 14.31; ESI-MS: 403 [M+H]^+^. Anal. Calcd for C_21_H_22_N_8_O: C, 62.67; H, 5.51; N, 27.84. Found: C, 62.65; H, 5.48; N, 27.81.

**3-(2,5-dimethyl-5H-imidazo[1,2-b]pyrazol-3-yl)-5-(1-(4-methoxyphenyl)-1H-1,2,3-triazol-4-yl)-1,2,4-oxadiazole (8e):** M.p: 156-158 ^o^C; ^1^H-NMR (400 MHz, DMSO-*d*_6_) δ 8.13 (s, 1H, tri-H),7.73 (d, *J* = 8.0 Hz, 1H), 7.69 (d, *J* = 8.0 Hz, 2H, Ar-H), 7.42 (d, *J* = 8.0 Hz, 1H), 7.00 (d, *J* = 8.0 Hz, 2H, Ar-H), 3.86 (s, 3H, O-CH_3_), 3.63 (s, 3H, N-CH_3_), 2.32 (s, 3H, -CH_3_); ^13^C-NMR (100 MHz, DMSO-*d*_6_) δ 170.70, 164.17, 160.55, 159.29, 139.40, 132.57, 131.35, 126.73(2C), 125.49, 118.56, 114.62(2C), 110.48, 104.33, 56.49, 36.62, 18.17; ESI-MS: 377 [M+H]^+^. Anal. Calcd for C_18_H_16_N_8_O_2_: C, 57.44; H, 4.29; N, 29.77. Found: C, 57.42; H, 4.26; N, 29.75.

**5-(1-(3,5-dimethoxyphenyl)-1H-1,2,3-triazol-4-yl)-3-(2,5-dimethyl-5H-imidazo[1,2-b]pyrazol-3-yl)-1,2,4-oxadiazole (8f):** M.p: 168-170 ^o^C; ^1^H-NMR (400 MHz, DMSO-*d*_6_) δ 8.10 (s, 1H, tri-H), 7.71 (d, *J* = 8.0 Hz, 1H), 7.61 (s, 2H, Ar-H), 7.40 (d, *J* = 8.0 Hz, 1H), 6.97 (s, 1H), 3.82 (s, 6H, Ar-OCH_3_), 3.61 (s, 3H, N-CH_3_), 2.31 (s, 3H, -CH_3_);^13^C-NMR (100 MHz, DMSO-*d*_6_) δ 170.29, 164.35, 160.53, 158.40(2C), 140.46, 139.30, 132.57, 125.58, 118.57, 110.40, 104.50, 102.18(2C), 98.60, 55.10(2C), 36.32, 18.45; ESI-MS: 407 [M+H]^+^. Anal. Calcd for C_19_H_18_N_8_O_3_: C, 56.15; H, 4.46; N, 27.57. Found: C, 56.11; H, 4.43; N, 27.54.

**5-(1-(4-chlorophenyl)-1H-1,2,3-triazol-4-yl)-3-(2,5-dimethyl-5H-imidazo[1,2-b]pyrazol-3-yl)-1,2,4-oxadiazole (8g):** M.p: 181-183 ^o^C; ^1^H-NMR (400 MHz, DMSO-*d*_6_) δ 8.13 (s, 1H, tri-H), 7.78 (d, *J* = 8.0 Hz, 2H, Ar-H), 7.72 (d, *J* = 8.0 Hz, 1H), 7.46 (d, *J* = 8.0 Hz, 2H, Ar-H), 7.41 (d, *J* = 8.0 Hz, 1H), 3.63 (s, 3H, N-CH_3_), 2.32 (s, 3H, -CH_3_); ^13^C-NMR (100 MHz, DMSO-*d*_6_) δ 170.56, 164.39, 160.69, 139.22, 136.80, 133.36, 132.64, 129.31(2C), 125.40, 124.18(2C), 118.73, 110.54, 104.46, 36.64, 18.44.; ESI-MS: 381 [M+H]^+^. Anal. Calcd for C_17_H_13_ClN_8_O: C, 53.62; H, 3.44; N, 29.43. Found: C, 53.59; H, 3.42; N, 29.41.

**3-(2,5-dimethyl-5H-imidazo[1,2-b]pyrazol-3-yl)-5-(1-(4-fluorophenyl)-1H-1,2,3-triazol-4-yl)-1,2,4-oxadiazole (8h):** M.p: 150-152 ^o^C; ^1^H-NMR (400 MHz, DMSO-*d*_6_) δ 8.18 (s, 1H, tri-H), 8.09 (d, *J* = 8.0 Hz, 2H, Ar-H), 7.81 (d, *J* = 8.0 Hz, 2H, Ar-H), 7.73 (d, *J* = 8.0 Hz, 1H), 7.42 (d, *J* = 8.0 Hz, 1H), 3.64 (s, 3H, N-CH_3_), 2.33 (s, 3H, -CH_3_); ^13^C-NMR (100 MHz, DMSO-*d*_6_) δ 170.48, 164.57, 161.62, 160.22, 159.56, 139.72, 133.71, 132.33, 125.48, 123.41, 123.35, 118.83, 117.84, 117.63, 110.76, 104.58, 36.35, 18.46; ESI-MS: 365 [M+H]^+^. Anal. Calcd for C_17_H_13_FN_8_O: C, 56.04; H, 3.60; N, 30.76. Found: C, 56.01; H, 3.57; N, 30.78.

**5-(1-(4-bromophenyl)-1H-1,2,3-triazol-4-yl)-3-(2,5-dimethyl-5H-imidazo[1,2-b]pyrazol-3-yl)-1,2,4-oxadiazole (8i):** M.p: 145-147 ^o^C; ^1^H-NMR (400 MHz, DMSO-*d*_6_) δ 8.11 (s, 1H, tri-H),7.73 (d, *J* = 8.0 Hz, 1H), 7.62 (d, *J* = 8.0 Hz, 2H, Ar-H), 7.54 (d, *J* = 8.0 Hz, 2H, Ar-H), 7.41 (d, *J* = 8.0 Hz, 1H), 3.63 (s, 3H, N-CH_3_), 2.33 (s, 3H, -CH_3_); ^13^C-NMR (100 MHz, DMSO-*d*_6_) δ 170.68, 164.32, 160.43, 139.35, 135.11, 132.23, 131.29(2C), 125.40, 123.69(2C), 120.58, 118.17, 110.82, 104.24, 36.31, 18.44; ESI-MS: 425 [M+H]^+^. Anal. Calcd for C_17_H_13_BrN_8_O: C, 48.02; H, 3.08; N, 26.35. Found: C, 48.00; H, 3.05; N, 26.38.

**5-(1-(3,5-dichlorophenyl)-1H-1,2,3-triazol-4-yl)-3-(2,5-dimethyl-5H-imidazo[1,2-b]pyrazol-3-yl)-1,2,4-oxadiazole (8j):** M.p: 161-163 ^o^C; ^1^H-NMR (400 MHz, DMSO-*d*_6_) δ 8.12 (s, 1H, tri-H),7.74 (d, *J* = 8.0 Hz, 1H), 7.66 (s, 2H, Ar-H), 7.42 (d, *J* = 8.0 Hz, 1H), 7.30 (s, 1H, Ar-H), 3.64 (s, 3H, N-CH_3_), 2.33 (s, 3H, -CH_3_); ^13^C-NMR (100 MHz, DMSO-*d*_6_) δ_C_ 170.51, 164.56, 160.58, 139.17, 138.15, 135.70(2C), 132.63, 125.38, 124.12, 122.45(2C), 118.79, 110.63, 104.35, 36.32, 18.45; ESI-MS: 415 [M+H]^+^. Anal. Calcd for C_17_H_12_Cl_2_N_8_O: C, 49.17; H, 2.91; N, 26.99. Found: C, 49.15; H, 2.88; N, 26.95.

**5-(1-(3,5-dibromophenyl)-1H-1,2,3-triazol-4-yl)-3-(2,5-dimethyl-5H-imidazo[1,2-b]pyrazol-3-yl)-1,2,4-oxadiazole (8k):** M.p: 160-162 ^o^C; ^1^H-NMR (400 MHz, DMSO-*d*_6_) δ8.14 (s, 1H, tri-H), 7.74 (d, *J* = 8.0 Hz, 1H), 7.58 (s, 2H, Ar-H), 7.41 (d, *J* = 8.0 Hz, 1H), 7.32 (s, 1H, Ar-H), 3.64 (s, 3H, N-CH_3_), 2.33 (s, 3H, -CH_3_);^13^C-NMR (100 MHz, DMSO-*d*_6_) δ 170.34, 164.54, 160.49, 139.50, 137.70, 132.58, 129.56, 127.73(2C), 125.44, 121.54(2C), 118.71, 110.52, 104.45, 36.65, 18.19; ESI-MS: 503 [M+H]^+^. Anal. Calcd for C_17_H_12_Br_2_N_8_O: C, 40.50; H, 2.40; N, 22.23. Found: C, 40.48; H, 2.37; N, 22.20.

**5-(1-(3,5-difluorophenyl)-1H-1,2,3-triazol-4-yl)-3-(2,5-dimethyl-5H-imidazo[1,2-b]pyrazol-3-yl)-1,2,4-oxadiazole (8l):** M.p: 169-171 ^o^C; ^1^H-NMR (400 MHz, DMSO-*d*_6_) δ 8.15 (s, 1H, tri-H), 8.08 (s, 2H, Ar-H), 7.84 (s, 1H, Ar-H), 7.73 (d, *J* = 8.0 Hz, 1H), 7.42 (d, *J* = 8.0 Hz, 1H), 3.64 (s, 3H, N-CH_3_), 2.32 (s, 3H, -CH_3_); ^13^C-NMR (100 MHz, DMSO-*d*_6_) δ 170.45, 164.37, 163.27, 163.20, 161.18, 161.11, 160.13, 139.43, 135.20, 135.13, 135.05, 132.34, 125.59, 118.64, 110.60, 106.55, 106.34, 106.14, 104.29, 103.46, 103.40, 103.19, 103.12, 36.61, 18.76; ESI-MS: 383 [M+H]^+^. Anal. Calcd for C_17_H_12_F_2_N_8_O: C, 53.41; H, 3.16; N, 29.31. Found: C, 53.38; H, 3.12; N, 29.29.

**3-(2,5-dimethyl-5H-imidazo[1,2-b]pyrazol-3-yl)-5-(1-(4-nitrophenyl)-1H-1,2,3-triazol-4-yl)-1,2,4-oxadiazole (8m):** M.p: 176-178 ^o^C; ^1^H-NMR (400 MHz, DMSO-*d*_6_) δ 8.28 (d, *J* = 8.0 Hz, 2H, Ar-H), 8.15 (s, 1H, tri-H), 8.06(d, *J* = 8.0 Hz, 2H, Ar-H), 7.75 (d, *J* = 8.0 Hz, 1H), 7.43 (d, *J* = 8.0 Hz, 1H), 3.64 (s, 3H, N-CH_3_), 2.33 (s, 3H, -CH_3_); ^13^C-NMR (100 MHz, DMSO-*d*_6_) δ 170.26, 164.49, 160.48, 146.33, 140.65, 139.45, 132.81, 126.78(2C), 125.28, 123.62(2C), 118.52, 110.67, 104.36, 36.29, 18.76; ESI-MS: 392 [M+H]^+^. Anal. Calcd for C_17_H_13_N_9_O_3_: C, 52.17; H, 3.35; N, 32.21. Found: C, 52.15; H, 3.33; N, 32.25.

**4-(4-(3-(2,5-dimethyl-5H-imidazo[1,2-b]pyrazol-3-yl)-1,2,4-oxadiazol-5-yl)-1H-1,2,3-triazol-1-yl)benzonitrile (8n):** M.p: 181-183 ^o^C; ^1^H-NMR (400 MHz, DMSO-*d*_6_) δ 8.13 (s, 1H, tri-H), 7.86 (d, *J* = 8.0 Hz, 2H, Ar-H), 7.73 (d, *J* = 8.0 Hz, 1H), 7.51 (d, *J* = 8.0 Hz, 2H, Ar-H), 7.40 (d, *J* = 8.0 Hz, 1H), 3.63 (s, 3H, N-CH_3_), 2.33 (s, 3H, -CH_3_); ^13^C-NMR (100 MHz, DMSO-*d*_6_) δ 170.33, 164.53, 160.54, 139.29, 138.27, 132.43, 128.77(2C), 126.64(2C), 125.54, 119.71, 118.56, 116.23, 110.45, 104.29, 36.64, 18.47; ESI-MS: 372 [M+H]^+^. Anal. Calcd for C_18_H_13_N_9_O: C, 58.22; H, 3.53; N, 33.95. Found: C, 58.19; H, 3.51; N, 33.99.

1H-NMR, 13C-NMR and Mass copies (Figure S1-

**Figure S1.** ^1^H-NMR of compound **3**

**Figure S2.** ^1^H-NMR of compound **6**

 **Figure S3.** ^1^H-NMR of compound **8a**

 **Figure S4.** ^13^C-NMR of compound **8a**

**Figure S5.** ^1^H-NMR of compound **8b**

**Figure S6.** ^13^C-NMR of compound **8b**

**Figure S7.** ^1^H-NMR of compound **8c**

**Figure S8.** ^13^C-NMR of compound **8c**

**Figure S9.** ^1^H-NMR of compound **8d**

**Figure S10.** ^13^C-NMR of compound **8d**

**Figure S11.** ^1^H-NMR of compound **8e**

**Figure S12.** ^13^C-NMR of compound **8e**

**Figure S13.** ^1^H-NMR of compound **8g**

**Figure S14.** ^13^C-NMR of compound **8g**

**Figure S15.** ^1^H-NMR of compound **8h**

**Figure S16.** ^13^C-NMR of compound **8h**

**Figure S17.** ^1^H-NMR of compound **8j**

**Figure S18.** ^13^C-NMR of compound **8j**

**Figure S19.** ^1^H-NMR of compound **8l**

**Figure S20.** ^13^C-NMR of compound **8l**

**Figure S21.** ESI-MS of compound **3**

 **Figure S22.** ESI-MS of compound **6**

**Figure S23.** ESI-MS of compound **8a**

**Figure S24.** ESI-MS of compound **8b**

**Figure S25.** ESI-MS of compound **8c**

**Figure S26.** ESI-MS of compound **8d**

**Figure S27.** ESI-MS of compound **8e**

**Figure S28.** ESI-MS of compound **8g**

**Figure S29.** ESI-MS of compound **8h**

**Figure S30.** ESI-MS of compound **8j**

**Figure S31.** ^1^H-NMR of compound **8l**
